# Accumulation of L-type Bovine Prions in Peripheral Nerve Tissues

**DOI:** 10.3201/eid1607.091882

**Published:** 2010-07

**Authors:** Yoshifumi Iwamaru, Morikazu Imamura, Yuichi Matsuura, Kentaro Masujin, Yoshihisa Shimizu, Yujing Shu, Megumi Kurachi, Kazuo Kasai, Yuichi Murayama, Shigeo Fukuda, Sadao Onoe, Ken’ichi Hagiwara, Yoshio Yamakawa, Tetsutaro Sata, Shirou Mohri, Hiroyuki Okada, Takashi Yokoyama

**Affiliations:** Author affiliations: National Institute of Animal Health, Tsukuba, Ibaraki, Japan (Y. Iwamaru, M. Imamura, Y. Matsuura, K. Masujin, Y. Shimizu, Y. Shu, M. Kurachi, K. Kasai, Y. Murayama, S. Mohri, H. Okada, T. Yokoyama);; Hokkaido Animal Research Center, Hokkaido, Japan (S. Fukuda, S. Onoe); and National Institute of Infectious Diseases, Tokyo, Japan (K. Hagiwara, Y. Yamakawa, T. Sata)

**Keywords:** atypical bovine spongiform encephalopathy, Creutzfeldt-Jacob disease, cattle, distribution, L-type, nerve tissues, prion, neurodegenerative disorder, transmissibility, zoonoses, dispatch

## Abstract

We recently reported the intraspecies transmission of L-type atypical bovine spongiform encephalopathy (BSE). To clarify the peripheral pathogenesis of L-type BSE, we studied prion distribution in nerve and lymphoid tissues obtained from experimentally challenged cattle. As with classical BSE prions, L-type BSE prions accumulated in central and peripheral nerve tissues.

Bovine spongiform encephalopathy (BSE) is a fatal neurodegenerative disorder of cattle characterized by accumulation of a protease-resistant form of a normal cellular prion protein (PrPres) in the central nervous system. The scientific literature in general has assumed that BSE in cattle is caused by a uniform strain (classical BSE). However, different neuropathologic and molecular phenotypes of BSE (atypical BSEs) have recently been reported from various countries (*1*). Recent data from Western blot analyses of field cases of atypical BSEs are characterized by a higher (H-type BSE) or lower (L-type BSE) molecular mass of the unglycosylated form of PrPres than is classical BSE (*2*). The origins of atypical BSEs remain obscure; unlike classical BSE, atypical BSE has been detected mainly in aged cattle and suggested a as possible sporadic form of BSE (*3*).

Several lines of evidence demonstrate that classical BSE and a variant form of Creutzfeldt-Jacob disease are most likely caused by the same agent (*4**,**5*). Transmission of classical BSE to humans has been proposed to result from ingestion of contaminated food. Whether atypical BSEs are transmissible to humans remains uncertain; however, human susceptibility to L-type BSEs is suggested by recent experimental transmission in primates (*6*) and mice transgenic for human prion protein (PrP) (*7*) by using the most effective route of intracerebral inoculations of prions. The L-type BSE prion is much more virulent in primates and in humanized mice than is the classical BSE prion, which suggests the possibility of zoonotic risk associated with the L-type BSE prion. These findings emphasize the critical importance of understanding tissue distribution of L-type BSE prions in cattle because, among the current administrative measures for BSE controls, the specified risk materials removal policy plays a crucial role in consumer protection.

In Japan, atypical BSE was detected in an aged Japanese Black cow (BSE/JP24) (*8*). We recently reported the successful transmission of BSE/JP24 prions to cattle and showed that the characteristics of these prions closely resemble those of L-type BSE prions found in Italy (*9*). In this study, we report the peripheral distribution of L-type BSE prions in experimentally challenged cattle.

## The Study

The Animal Ethics Committee and Animal Care and Use Committee of the National Institute of Animal Health approved the study. Five Holstein calves 2–3 months of age were intracerebrally injected with 1 mL of 10% (w/v) brain homogenates prepared from the medulla oblongata of BSE/JP24. In our earlier report, experimentally challenged cattle appeared to display clinical signs indicative of BSE at 11 months postinoculation (mpi) (*9*). Animals were sequentially euthanized before and after the onset of clinical signs (cattle identification codes 8515 and 496 at 10 and 12 mpi, respectively) and at the terminal stage of the disease (cattle identification codes 528, 1061, and 5566 at 16 mpi). A wide range of tissues was sampled at subsequent necropsy. We provisionally categorized the adrenal gland as nerve tissue because of the presence of chromaffin cells in the medulla of the gland.

Western blot analysis for PrPres was performed on obex tissue samples as described previously by using anti-PrP monoclonal antibody T2 (*9*). PrPres was detectable in all obex samples obtained 10, 12, and 16 mpi, suggesting that transmission of L-type BSE prions to these animals was successful. Dilution of the protease-treated brain sample and analysis of Western blot results showed that the detection threshold for PrPres was 1.25 μg of brain tissue equivalent (data not shown).

**Figure 1:**
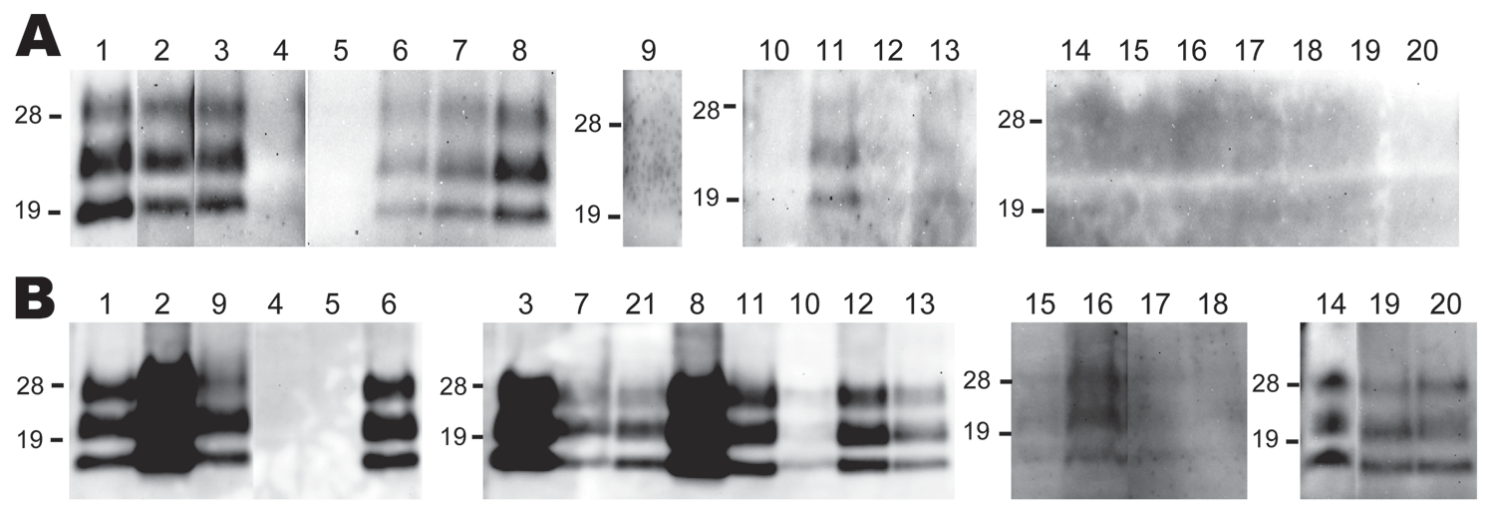
Western blot analysis of a protease-resistant form (PrPres) of a normal cellular prion protein in nerve tissue samples obtained from cattle 10 (A) and 16 (B) months postinoculation (cattle identification codes 8515 and 1061, respectively). The nerve tissues tested are shown above the lanes: 1, trigeminal ganglia; 2, pituitary gland; 3, anterior cervical ganglion; 4, facial nerve; 5, hypoglossal nerve; 6, cranial mesenteric ganglia; 7, vagus nerve (cervical part); 8, stellate ganglia; 9, adrenal gland; 10, phrenic nerve; 11, vagus nerve (pectoral part); 12, vagosympathic trunk (pectoral part); 13, vagosympathetic trunk (lumbar part); 14, accessory nerve; 15, suprascapular nerve; 16, brachial nerve plexus; 17, median nerve; 18, radial nerve; 19, sciatic nerve; 20, tibial nerve, 21, middle cervical ganglion. The equivalent of 100 mg of tissue was loaded. Western blots were probed with monoclonal antibody T2 to detect PrPres. Molecular mass standards (kDa) are indicated on the left of each panel.

A variety of nerve and lymphoid tissue samples were investigated for accumulation of PrPres by Western blot analysis by using phosphotungstic acid precipitation, as described previously (*10*); examples of cattle tissue samples obtained 10 and 16 months mpi (codes 8515 and 1061, respectively) are shown in Figure 1. In cattle at the preclinical stage, PrPres was detectable in all tested ganglia and barely detectable in the vagus nerve and vagosympathic trunk. In cattle at the terminal stage, PrPres was barely detectable in the forelimb nerves (suprascapular nerve, brachial nerve plexus, median nerve, and radial nerve), whereas substantial amounts of PrPres were present in other nerve tissues except for facial and hypoglossal nerves (Table). A broader nerve tissue distribution of PrPres was observed in cattle at 16 mpi than at 10 and 12 mpi. Contrary to what we found in nerve tissues, we detected no PrPres from tests performed on lymphoid tissues obtained from any of the 5 cattle studied.

**Table:** Western blot detection of PrPres in tissue samples obtained from cattle intracerebrally challenged with BSE/JP24 prion*

Tissue samples	Cattle identification codes

8515 (10 mpi)	498 (12 mpi)	528 (16 mpi)	1061 (16 mpi)	5566 (16 mpi)
Nerve tissues					
Obex	+	+	+	+	+
Spinal cord	+	+	+	+	+
Cauda equina	+	+	+	+	+
Optic nerve	+	+	+	+	+
Pituitary gland	+	+	+	+	+
Trigeminal ganglia	+	+	+	+	+
Cranial cervical ganglia	+	+	+	+	+
Stellate ganglia	+	+	+	+	+
Vagosympathic trunk	+	+	+	+	+
Cranial mesenteric ganglia	+	+	+	+	+
Vagus nerve	+	+	+	+	+
Facial nerve	−	−	−	−	−
Hypoglossal nerve	−	−	−	−	−
Phrenic nerve	−	+	+	+	+
Accessory nerve	−	+	+	+	+
Suprascapular nerve	−	−	+	+	+
Brachial nerve plexus	−	−	+	+	+
Median nerve	−	−	+	+	−
Radial nerve	−	−	+	+	−
Sciatic nerve	−	+	+	+	+
Tibial nerve	−	+	+	+	+
Adrenal gland	−	+	+	+	+
Lymphoid tissues					
Spleen	−	−	−	−	−
Tonsil	−	−	−	−	−
Parotid lymph nodes	−	−	−	−	−
Lateral retropharyngeal lymph nodes	−	−	−	−	−
Mandibular lymph nodes	−	−	−	−	−
Brachiocephalic lymph node	−	−	−	−	−
Anterior cervical lymph node	−	−	−	−	−
Axillary lymph nodes	−	−	−	−	−
Superficial inguinal lymph nodes	−	−	−	−	−
Subiliac lymph nodes	−	−	−	−	−
Popliteal lymph nodes	−	−	−	−	−
Splenic lymph nodes	−	−	−	−	−
Hepatic lymph nodes	−	−	−	−	−
Internal iliac lymph nodes	−	−	−	−	−
External iliac lymph nodes	−	−	−	−	−
Mesenteric lymph nodes	−	−	−	−	−

*PrPres, prion protein resistant; BSE, bovine spongiform encephalopathy; mpi, months postinoculation; +, positive for PrPres; –, negative for PrPres.

**Figure 2:**
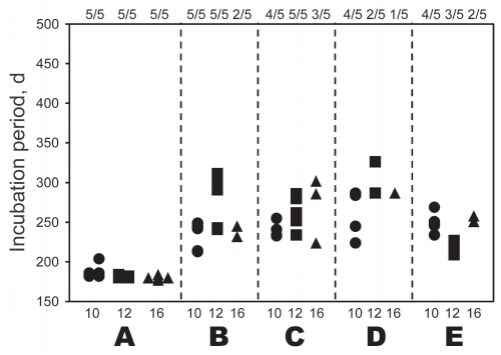
Bioassay using nerve tissues obtained from bovine spongiform encephalopathy JP24 prion-inoculated cattle. Inocula from selected tissues—obex (A), sciatic nerve (B), adrenal gland (C), branchial nerve plexus (D), and vagus nerve cervical part (E)—were prepared from cattle euthanized at 10 (code 8515, circle), 12 (code 498, square), and 16 (code 5566, triangle) months postinoculation were and inoculated intracerebrally into mice transgenic for bovine prion protein. Ratios above graph indicate number of prion-diseased mice/number of inoculated mice at 500 d postinoculation.

Infectivity of selected nerve tissues (including the obex, sciatic nerve, adrenal gland, brachial nerve plexus, and vagus nerve) obtained from cattle euthanized at 10, 12, and 16 mpi (codes 8515, 498, and 5566, respectively) was analyzed by intracerebral injection into mice transgenic for bovine prion protein, as described previously (*11*). As a negative control, mice were injected with cells from the brainstem of a normal cow. The presence of PrPres in the brains of all mice used in the experiment was determined by Western blot analysis. Infectivity was detected in all nerve tissues tested, regardless of the presence of detectable PrPres (Figure 2). Control mice showed no apparent abnormality >500 days postinoculation.

## Conclusions

We report accumulation of L-type atypical BSE prions in peripheral nerve tissues sampled from intracerebrally challenged cattle. Our study demonstrated that almost all of the peripheral nerve tissues tested became PrPres positive in a time-dependent manner, whereas no PrPres was detectable in lymphoid tissues, even in cattle with fatal atypical BSE. Our results suggest the possibility that, like classical BSE prions, L-type BSE prions propagated in the central nervous system and were spread centrifugally by nerve pathways (*11**,**12*). In Italy, L-type BSE prions have been characterized in detail by using cattle challenged intracerebrally. However, PrPres was not detected in their peripheral tissues, including the peripheral nerves (*13*). The reason for the discrepancy in PrPres detection is unclear. In view of the similarities between the L-type and BSE/JP24 prion characteristics (*9*), this discrepancy may result from differences in the methods used for PrPres detection.

We detected infectivity in the nerve tissue samples (including samples from the obex, sciatic nerve, adrenal gland, brachial nerve plexus, and vagus nerve) obtained 10, 12, and 16 mpi. On the basis of the incubation time of 223 ± 25 (mean ± SD) days in mice injected with a 1,000-fold dilution of the obex homogenate, infectious titers in peripheral nerve tissues appeared to be 1,000 × lower than those estimated in the obex during endpoint titration of infectivity.

Our results demonstrate that L-type atypical BSE prions can be distributed in the peripheral nerve tissues of intracerebrally challenged cattle. These findings are useful for understanding L-type BSE pathogenesis and accurately assessing the risks associated with this disease. Investigations of prion distribution in cattle that have been orally challenged with L-type BSE prions are critical.
